# Development and interpretable machine learning models for classification of pancreatic pseudocyst risk in acute pancreatitis

**DOI:** 10.3389/fdgth.2026.1753529

**Published:** 2026-03-17

**Authors:** Hailong Feng, Ping Wang, Weihan He, Liwei Shang, Mingrui Cui, Keyang Wang, Kejia An, Yingjian Zhang

**Affiliations:** 1 Department of Gastroenterology, The First Affiliated Hospital, College of Clinical Medicine, Henan University of Science and Technology, Luoyang, Henan, China; 2 Henan Medical Key Laboratory of Gastrointestinal Microecology and Hepatology, Luoyang, Henan, China; 3 College of Basic Medicine and Forensic Medicine, Henan University of Science and Technology, Luoyang, Henan, China

**Keywords:** acute pancreatitis, machine learning, pancreatic pseudocyst, risk classification, SHAP, temporal validation

## Abstract

**Introduction:**

Pancreatic pseudocysts (PPC) are a late local complication of acute pancreatitis (AP). Persistent PPC carry a high risk of severe outcomes. Existing models, which are predominantly based on logistic regression, exhibit limited predictive performance and have not undergone temporal validation. This study aimed to develop and validate an interpretable machine learning model using routinely available clinical data for classifying AP patients according to PPC development status.

**Materials and methods:**

We retrospectively analyzed 1,184 AP patients admitted to a tertiary hospital between 2018 and 2023. Data from 2018 to 2022 (*n* = 979) were randomly split into training (70%, *n* = 685) and internal test (30%, *n* = 294) sets, while the 2023 cohort (*n* = 205) served as an independent temporal validation set. Candidate predictors—including demographic characteristics, clinical history, and routine laboratory parameters—were screened via univariate analysis and further selected using LASSO regression to address multicollinearity. Nine machine learning algorithms were developed and compared: logistic regression, decision tree, random forest, artificial neural network, support vector machine, K-nearest neighbors, naïve Bayes, XGBoost, and LightGBM. Model performance was evaluated using area under the curve (AUC), accuracy, sensitivity, specificity, positive predictive value and negative predictive value. The optimal model was interpreted using SHapley Additive exPlanations (SHAP).

**Results:**

LASSO regression selected seven predictors: diabetes history, pancreatitis history, biliary surgery history, C-reactive protein, albumin, blood urea nitrogen, and serum calcium. The random forest model demonstrated the best classification performance, achieving an AUC of 0.884 (95% CI: 0.827–0.941) on the internal test set and 0.914 (95% CI: 0.845–0.983) on the temporal validation set. SHAP analysis identified serum calcium and C-reactive protein as the most important predictors, with low calcium and elevated CRP substantially increasing the probability of PPC classification.

**Discussion:**

We developed and temporally validated interpretable machine learning models for classifying PPC development status using seven routinely available clinical indicators. The random forest model demonstrated excellent discrimination and generalizability, while SHAP analysis provided transparent explanations of individual classifications. These models may facilitate early identification of high-risk AP patients and guide proactive clinical management.

## 1 Introduction

Acute pancreatitis (AP) is a common digestive system emergency, with a global incidence of approximately 34 per 100,000 and rising, making it a significant public health issue (1, 2). About 20% of AP patients progress to moderately severe or severe acute pancreatitis, characterized by persistent organ failure and/or local complications, with a mortality rate as high as 20%–40% (3). Pancreatic pseudocyst (PPC) is an encapsulated collection of fluid rich in pancreatic enzymes but lacking solid necrotic tissue, formed by fibrous or granulation tissue following AP or pancreatic injury (4). It usually matures 4 weeks after the acute episode and is a late local complication of AP, often overlooked by discharged patients due to lack of attention (5). Current studies have found its incidence in AP to be 10%–20%, of which 33%–40% may resolve spontaneously (6). However, for pseudocysts larger than 6 cm in diameter and persisting for more than 6 weeks, they are generally considered indications requiring active intervention. Current intervention methods mainly include percutaneous drainage, endoscopic drainage, and surgical procedures (7). If not detected and intervened in a timely manner, serious complications such as infection, bleeding, rupture, or compression of adjacent organs are highly likely, thereby affecting patient prognosis (5).

The diagnosis of PPC primarily relies on imaging examinations such as ultrasound, CT, and MRI. However, these methods can only detect already matured PPCs and cannot provide early prediction for this late complication (3). Chen et al. identified ESR, PLT, and Hb as independent predictors of pancreatic pseudocysts through logistic regression analysis and further constructed a three-parameter combined prediction system (8). Hou et al. developed a nomogram based on medical history and complete blood count results, including a history of severe pancreatitis, diabetes, biliary surgery history, hemoglobin, albumin, and body mass index. It showed good predictive value with an AUC of 0.883 in the training set and 0.839 in the internal test set (5). Although indicators assessing the severity of acute pancreatitis have some guiding significance for the early prevention and management planning of pseudocysts. Ji et al. developed a nomogram integrating early laboratory indicators (LDH, Hb, ALB, and Ca) that demonstrated higher accuracy in predicting PPC occurrence compared to traditional scores like the Balthazar CT severity index (CTSI) and AP severity (RAC), providing a more effective tool for early identification of high-risk patients in clinical practice (6). It is worth noting that these logistic regression-based models have not undergone temporal validation and have not been widely applied in clinical practice.

Machine learning, as an interdisciplinary field that learns patterns from known data to predict unknown data, encompasses common algorithms such as logistic regression, decision trees, random forests, artificial neural networks, support vector machines, K-nearest neighbors, naive Bayes, XGBoost, and LightGBM (9). In recent years, machine learning has shown significant advantages in medical prediction models, particularly in integrating multidimensional clinical data (such as laboratory indicators, medical history, and imaging scores) and capturing complex nonlinear relationships among variables (10, 11). Current research on predicting pancreatic pseudocysts still primarily employs logistic regression models, which are suitable for scenarios with low event probability and approximately linear relationships between variables, effectively revealing linear associations between predictors and outcomes (12). However, a growing body of research suggests that complex machine learning models offer greater advantages in clinical prediction. For example, Zhang et al. used XGBoost to predict acute respiratory distress syndrome complicating acute pancreatitis, achieving an AUC of 0.93 in the validation set, significantly higher than the 0.85 of logistic regression (13). Ning et al. compared 10 machine learning models for predicting mortality in patients with infected pancreatic necrosis, with the random survival forest model performing best (14). Although machine learning models are often seen as “black boxes,” making their decision-making process difficult to interpret intuitively, techniques like SHAP can quantify each feature's contribution to the prediction outcome, thereby enhancing the transparency and trustworthiness of ML models in clinical practice (15).

This study aimed to develop and validate an interpretable machine learning model using routinely available clinical data for classifying AP patients according to PPC development status. Clinical data from AP patients admitted to the First Affiliated Hospital of Henan University of Science and Technology between 2018 and 2022 were retrospectively collected and randomly split into training and test sets in a 7:3 ratio. Key predictor variables were first screened in the training set through univariate analysis and LASSO regression to eliminate irrelevant or collinear variables (16). Based on the selected variables, nine commonly used machine learning models were trained, including logistic regression, decision tree, random forest, artificial neural network, support vector machine, K-nearest neighbors, naive Bayes, XGBoost, and LightGBM. To further assess model generalizability, data from 2023 were used as an temporal validation set. The best-performing model was selected based on comprehensive evaluation of multiple performance metrics including area under the receiver operating characteristic curve (AUC), accuracy, sensitivity, specificity, positive predictive value (PPV), and negative predictive value (NPV). Subsequently, SHAP analysis was applied to the optimal model to enhance interpretability for clinical decision-making.

## 2 Materials and methods

### 2.1 Data collection

This retrospective study utilized data from the electronic medical record system of the First Affiliated Hospital of Henan University of Science and Technology. Clinical information was collected for the first-admission acute pancreatitis (AP) patients between January 2018 and December 2023. Specifically, initial clinical indicators were collected within the first 24 h after admission. This study adhered to the guidelines of the Declaration of Helsinki and was approved by the Ethics Committee of the First Affiliated Hospital of Henan University of Science and Technology (Approval No: K-2025-B086). Given the retrospective nature of the study, informed consent was waived.

**Figure 1:**
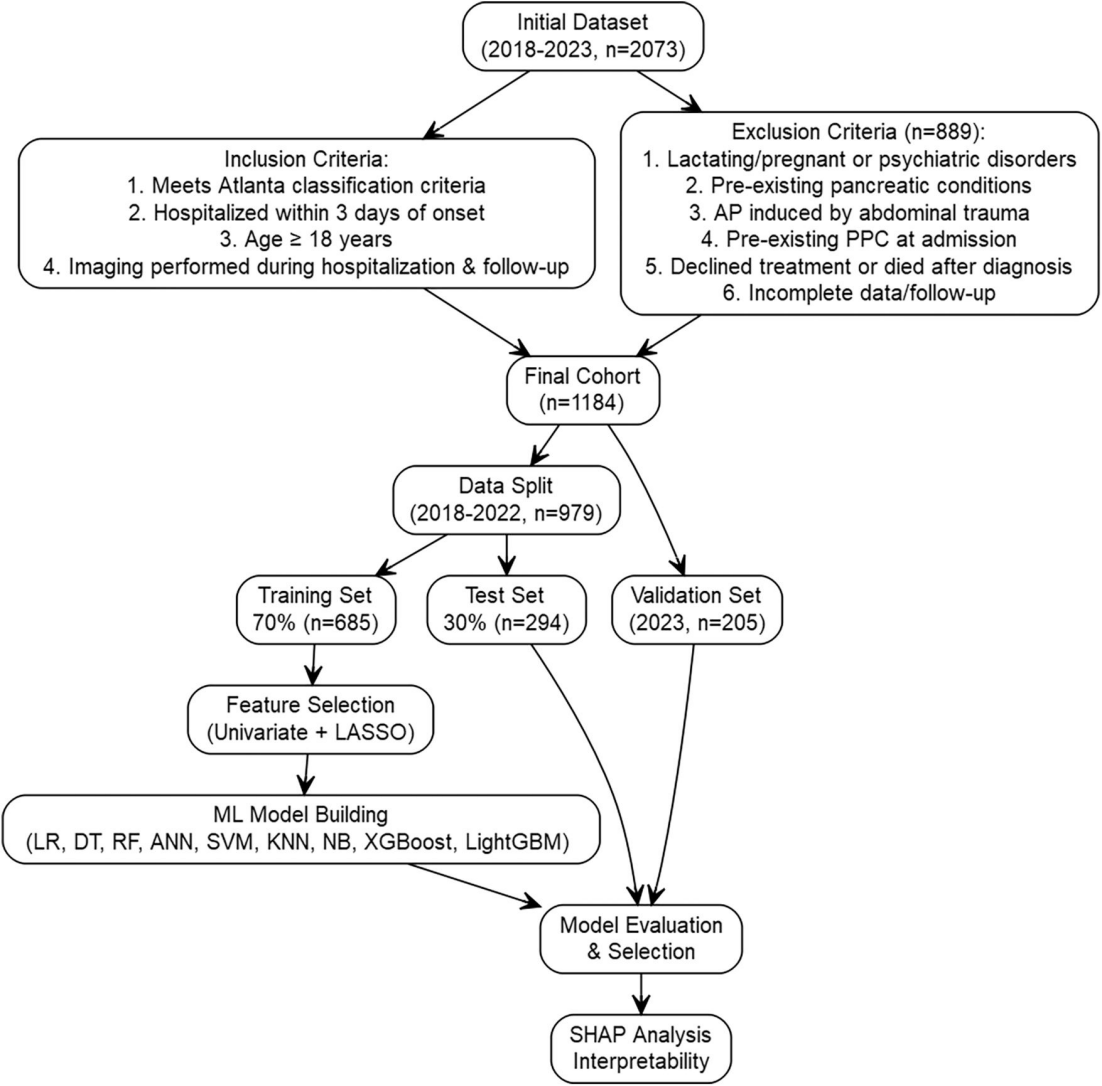
Study flowchart.

Inclusion criteria (1): Diagnosis of AP according to the Revised Atlanta Classification 2012 (4), requiring at least 2 of the following: a. Sudden onset of acute, persistent severe epigastric pain; b. Serum amylase and/or lipase levels >3 times the upper limit of normal; c. Characteristic imaging findings (abdominal ultrasound, CT, or MRI) consistent with AP (2); Hospital admission within 3 days of symptom onset; (3) Age ≥18 years; (4) Abdominal imaging was performed during hospitalization and the follow-up period to assess for PPC. PPC diagnosis was based on a history of AP, persistent encapsulated fluid collection for ≥4 weeks, characteristic imaging appearance, and exclusion of other pancreatic cystic lesions, chronic pancreatitis-related changes, or traumatic fluid collections (4, 17). All patients were followed up for at least 1 year after discharge to determine the presence of PPC, with diagnosis confirmed by abdominal imaging. Exclusion criteria: (1) Lactating or pregnant women, or patients with a history of psychiatric disorders; (2) Pre-existing pancreatic conditions, including pancreatic cystic diseases, pancreatic tumors, history of pancreatic trauma or surgery, or acute exacerbation of chronic pancreatitis; (3) AP induced by abdominal trauma; (4) Pre-existing PPC at admission; (5) Patients who declined treatment or died after diagnosis; (6) Incomplete clinical data, imaging studies, or clinical follow-up. Patients meeting any of the above criteria were excluded. After applying the inclusion and exclusion criteria, 889 patients were excluded, resulting in a final cohort of 1184 patients. Data from 2018 to 2022 (*n* = 979) were randomly split 7:3 into a training set (*n* = 685) and a test set (*n* = 294). Data from 205 patients in 2023 served as an temporal validation set to assess generalization. The study flowchart is shown in Figure 1.

### 2.2 Data collection

A total of 45 clinical variables were systematically collected, covering three major categories: demographic characteristics, clinical history, and laboratory parameters. Demographic characteristics included age, gender, height, weight, and body mass index (BMI). Clinical history comprised etiology, diabetes, hypertension, pancreatitis history (a documented history of acute pancreatitis in the admission record), biliary surgery history (including cholecystectomy or endoscopic biliary procedures), drinking history, smoking history, ascites, pleural effusion, days since onset, and length of hospital stay. Initial laboratory parameters, collected within the first 24 h after admission, included C-reactive protein (CRP), white blood cell count (WBC), absolute neutrophil count (N) and percentage (N/W), red blood cell count (RBC), hemoglobin (Hb), platelet count (PLT), amylase (AMY), lipase (LiP), glucose (GLU), total cholesterol (CHO), triglycerides (TG), total protein (TP), albumin (ALB), globulin (GLO), total bilirubin (TBIL), direct bilirubin (DBIL), indirect bilirubin (IBIL), alkaline phosphatase (ALP), lactate dehydrogenase (LDH), alanine aminotransferase (ALT), aspartate aminotransferase (AST), blood urea nitrogen (BUN), serum creatinine (SCr), potassium (K), sodium (Na), calcium (Ca), fibrinogen (FIB), and D-dimer.

### 2.3 Statistical analysis

#### 2.3.1 Data preprocessing

This study was designed from the outset to utilize basic medical record data available at admission and routine laboratory tests essential for the diagnosis of acute pancreatitis to construct the model, thereby ensuring its clinical practicality and ease of use. Consequently, variables with high missingness rates in clinical practice were excluded (8). For the absence of individual variables in a few patients, this study adopted the approach of directly deleting the data of this patient for processing. Variable assignment was conducted as follows. Etiology was classified in accordance with the 2013 American College of Gastroenterology (ACG) guidelines and categorized as Biliary (coded as 1), Hyperlipidemic (2), Alcoholic (3), and Other (4) (18). Binary variables—including gender (Male = 1, Female = 0), hypertension, pancreatitis history, biliary surgery history, drinking history, smoking history, ascites, and pleural effusion—were uniformly encoded as No = 0 and Yes = 1. The time from symptom onset to admission was stratified into three intervals: 0–24 h (coded as 1), 25–48 h (2), and 49–72 h (3); other continuous variables (e.g., laboratory measurements) were retained as original values.

#### 2.3.2 Feature selection

R software (version 4.4.1) was used for data processing and statistical analysis. To ensure reproducibility, a fixed random seed (“set.seed(1)”) was set prior to any random procedure, including dataset splitting and cross-validation. All categorical variables were converted to factors. Univariate analysis was first performed on the training set: normality of continuous variables was assessed using the Shapiro–Wilk test. Normally distributed data are expressed as Mean ± Standard Deviation (Mean ± SD) and compared using independent samples *t*-test; non-normally distributed variables are expressed as median and interquartile range [Median (Q1, Q3)] and compared using -Mann–Whitney *U*-test. Count data are expressed as frequency and percentage [n (%)] and compared using *χ*^2^-test. Variables with *P* < 0.05 in the univariate analysis were retained as candidate predictors. To further select features and address multicollinearity, LASSO regression was applied to these candidates. Continuous variables were normalized to the range [0,1] using min-max scaling, with the normalization parameters derived exclusively from the training set. The optimal penalty parameter *λ* was determined via 20-fold cross-validation using the “lambda.1se” criterion (i.e., the largest *λ* within one standard error of the minimum cross-validated error). Variables with non-zero coefficients at this optimal *λ* were selected for final model construction. This selection process was confined entirely to the training set to avoid data leakage. Key R packages used include “caret”, “glmnet”, “pROC” and “dplyr”.

#### 2.3.3 Model development

Multiple ML models (LR, DT, RF, KNN, SVM, NB, ANN, XGBoost, LightGBM) were built on the training set using the selected predictors. Table 1 summarizes the hyperparameter optimization strategies and search spaces for each classifier. Hyperparameter optimization was performed exclusively within the training set via 5-fold stratified cross-validation. Systematic search strategies—grid search or random search—were employed for each algorithm, except for logistic regression which served as a non-regularized linear baseline. Logistic regression (LR) was fitted using the glm function in R with family = binomial, employing maximum likelihood estimation. Decision trees (DT) were built with the rpart package, tuning the complexity parameter cp over {0.001, 0.005, 0.01, 0.05, 0.1}; the best cp = 0.01 was selected. Random forest (RF) implemented in randomForest optimized mtry (number of predictors sampled at each split, range 2–7) and ntree (number of trees, 100–500) via random search, yielding final values of mtry = 3 and ntree = 200. A single-hidden-layer artificial neural network (ANN) was built with the nnet package; a grid search over hidden units size (3, 5, 7, 9) and weight decay decay (0.01, 0.1, 0.5, 1.0) selected size = 5 and decay = 0.1 (max iterations = 200). Support vector machine (SVM) with a radial basis function kernel (e1071 package) was tuned via grid search over cost C (2⁻^2^, 2⁻^1^, 2^0^, 2^1^, 2^2^) and kernel parameter *γ* (2⁻^2^, 2⁻^1^, 2^0^, 2^1^, 2^2^), resulting in C = 1 and *γ* = 0.5. K-nearest neighbors (KNN) used the knn3 function from caret; the number of neighbors k was optimized over {3,5,7,9,11,13,15}, with the best k = 5. Naïve Bayes (NB) was implemented with Laplace smoothing (laplace = 1) and a grid search over the bandwidth adjustment factor adjust (0.5, 1.0, 1.5), which selected adjust = 1.0. For XGBoost, random search optimized learning rate eta (0.01, 0.1, 0.3), maximum tree depth max_depth (3, 5, 7), subsample ratio subsample (0.8, 1.0), and column subsample ratio colsample_bytree (0.8, 1.0); early stopping (early_stopping_rounds = 10) was applied with nrounds = 100, and the final parameters were eta = 0.1, max_depth = 5, subsample = 1.0, colsample_bytree = 1.0. LightGBM was similarly tuned via random search over learning_rate (0.01, 0.1, 0.2), num_leaves (15, 31, 63), feature_fraction (0.8, 1.0), and bagging_fraction (0.8, 1.0), with early stopping; the optimal combination was learning_rate = 0.1, num_leaves = 31, feature_fraction = 1.0, bagging_fraction = 1.0. All hyperparameter searches were conducted solely on the training set within the 5-fold cross-validation loop; the final models were retrained on the full training set using the best-found parameters and then evaluated on the test set and temporal validation set (19, 20).

**Table 1:** Summarizes the hyperparameter optimization strategies and search spaces for each classifier.

Classifier	Optimization	Hyperparameter	Search_space	Selected_value
LR	baseline	–	–	–
DT	Grid search (5-fold CV)	cp	{0.001,0.005,0.01,0.05,0.1}	0.01
RF	Random search (5-fold CV)	Mtry	2–7	3
		ntree	100–500	200
ANN	Grid search (5-fold CV)	Size	{3,5,7,9}	5
		decay	{0.01,0.1,0.5,1.0}	0.1
SVM	Grid search (5-fold CV)	C	{2^−2^,2^−1^,2^0^,2^1^,2^2^}	1
		*γ*	{2^−2^,2^−1^,2^0^,2^1^,2^2^}	0.5
KNN	Grid search (5-fold CV)	k	{3,5,7,9,11,13,15}	5
NB	Grid search (5-fold CV)	adjust	{0.5,1.0,1.5}	1.0
XGBoost	Random search (5-fold CV)	Eta	{0.01,0.1,0.3}	0.1
		max_depth	{3,5,7}	5
		Subsample	{0.8,1.0}	1.0
		colsample_bytree	{0.8,1.0}	1.0
LightGBM	Random search (5-fold CV)	learning_rate	{0.01,0.1,0.2}	0.1
		num_leaves	{15,31,63}	31
		feature_fraction	{0.8,1.0}	1.0
		bagging_fraction	{0.8,1.0}	1.0

#### 2.3.4 Model evaluation

Model performance was comprehensively assessed using multiple metrics, including area under the receiver operating characteristic curve (AUC), accuracy, sensitivity, specificity, positive predictive value (PPV), and negative predictive value (NPV). These metrics were calculated for all nine machine learning models across the training set, internal test set, and temporal validation set. The best-performing model was selected based on a comprehensive evaluation of these performance indicators. Calibration curves were also plotted to evaluate the agreement between predicted probabilities and observed outcomes. For the optimal model, interpretability analysis was performed on the test set using the SHAP (SHapley Additive exPlanations) framework. SHAP dependence plots illustrated the relationship between individual feature values and their impact on predictions, while SHAP summary plots (bee swarm and bar plots) provided global rankings of feature importance. Individual prediction explanations were generated to visualize the key risk drivers for specific patients. The Youden index was applied to the test set to determine the optimal probability threshold. All analyses were conducted using R (version 4.4.1). Key packages included “pROC” for ROC analysis and AUC calculation, “shapviz” and “fastshap” for SHAP interpretability, “caret” for performance metrics and confusion matrices, and “rms” and “CalibrationCurves” for calibration curve analysis.

## 3 Results

### 3.1 Characteristics of the study cohorts

After applying the inclusion and exclusion criteria, a total of 1,184 patients with acute pancreatitis were included in the final analysis. Among these, 148 patients (12.5%) developed pancreatic pseudocysts (PPC), while 1,036 patients (87.5%) did not. All patients were divided into three cohorts: a training set (*n* = 685), an internal test set (*n* = 294), and a temporal validation set (*n* = 205). Table 2 presents the baseline demographic and clinical characteristics of patients in the training set, stratified by PPC development status. In the training set, 87 patients (12.7%) developed PPC, while 598 patients (87.3%) did not. Statistical analysis showed significant differences (*P* < 0.05) between the two groups in 19 variables: Diabetes, Pancreatitis History, Biliary Surgery History, Drinking History, Smoking History, Length of Hospital Stay, CRP, N/W, GLU, TP, ALB, TBIL, IBIL, ALP, LDH, BUN, Ca, FIB, and D-Dimer. No significant differences (*P* > 0.05) were found in the remaining variables.

**Table 2:** Characteristics of the study cohorts.

Variables	Total patients (*n* = 685)	Non-pseudocyst (*n* = 598)	Pseudocyst (*n* = 87)	*P-*value
	*n* (%)	*n* (%)	*n* (%)	
Etiology				0.737
1	252 (37)	216 (36)	36 (41)	
2	251 (37)	219 (37)	32 (37)	
3	30 (4)	27 (5)	3 (3)	
4	152 (22)	136 (23)	16 (18)	
Gender				0.958
0	234 (34)	205 (34)	29 (33)	
1	451 (66)	393 (66)	58 (67)	
Diabetes				<0.001*
0	539 (79)	488 (82)	51 (59)	
1	146 (21)	110 (18)	36 (41)	
Hypertension				0.583
0	539 (79)	473 (79)	66 (76)	
1	146 (21)	125 (21)	21 (24)	
Pancreatitis_History				0.023*
0	547 (80)	486 (81)	61 (70)	
1	138 (20)	112 (19)	26 (30)	
Biliary_Surgery_History				<0.001*
0	624 (91)	554 (93)	70 (80)	
1	61 (9)	44 (7)	17 (20)	
Drinking_History				0.036*
0	508 (74)	452 (76)	56 (64)	
1	177 (26)	146 (24)	31 (36)	
Smoking_History				0.036*
0	522 (76)	464 (78)	58 (67)	
1	163 (24)	134 (22)	29 (33)	
Ascites				0.394
0	395 (58)	349 (58)	46 (53)	
1	290 (42)	249 (42)	41 (47)	
Pleural_Effusion				0.083
0	476 (69)	423 (71)	53 (61)	
1	209 (31)	175 (29)	34 (39)	
Days_Since_Onset				0.091
1	374 (55)	336 (56)	38 (44)	
2	204 (30)	172 (29)	32 (37)	
3	107 (16)	90 (15)	17 (20)	

Variables	Total patients (*n* = 685)	Non-pseudocyst (*n* = 598)	Pseudocyst (*n* = 87)	*P-*value
	“Mean ± SD” or “Median (IQR)”	“Mean ± SD” or “Median (IQR)”	“Mean ± SD” or “Median (IQR)”	
Length_of_Hospital_Stay, days	9 (5)	8 (5)	10 (5)	<0.001*
Age, years	42 (25)	43 (26)	40 (20)	0.177
Height, cm	170 (12)	170 (12)	170 (11)	0.351
Weight, kg	70 (19)	70 (20)	70 (15)	0.429
BMI, kg/m^2^	24.54 (5.04)	24.5 (5.28)	24.84 (3.02)	0.155
CRP, mg/L	107.03 (94.89)	106.72 (98.77)	107.45 (94.67)	<0.001*
WBC, ×10^9^/L	11.01 (7.09)	10.88 (6.96)	12.33 (7.35)	0.144
N, ×10^9^/L	9.4 (6.15)	9.2 (5.99)	10.36 (6.85)	0.083
N/W, %	0.85 (0.07)	0.85 (0.07)	0.87 (0.05)	0.004*
RBC, ×10^12^/L	4.54 (0.89)	4.54 (0.89)	4.48 (0.97)	0.486
Hb, g/L	141.58 ± 23.95	141.78 ± 23.65	140.22 ± 26.04	0.598
PLT, ×10^9^/L	220 (95)	219 (94)	233 (95)	0.617
AMY, U/L	533 (281)	534 (287.75)	528 (217.5)	0.893
LiP, U/L	875 (478)	872 (457.75)	914.5 (549.3)	0.52
GLU, mmol/L	8.21 (5.73)	7.94 (5.42)	9.52 (6.77)	0.002*
CHO, mmol/L	6.52 (3.79)	6.55 (3.83)	6.07 (3.34)	0.617
TG, mmol/L	1.98 (4.27)	1.96 (3.88)	2.72 (4.81)	0.758
TP, g/L	64.6 ± 7.77	64.85 ± 7.73	62.88 ± 7.87	0.031*
ALB, g/L	37.99 ± 5.66	38.38 ± 5.66	35.3 ± 4.89	<0.001*
GLO, g/L	26.3 (6.4)	26.1 (6.4)	27.4 (5.55)	0.062
TBIL, μmol/L	15 (18.8)	14.8 (18.77)	17.9 (16.3)	0.017*
DBIL, μmol/L	5.3 (7.2)	5.1 (7.37)	6 (5.55)	0.188
IBIL, μmol/L	11.1 (10.4)	10.7 (10.4)	12.8 (7.7)	0.009*
ALP, U/L	111 (84)	108.5 (84.5)	116 (68)	0.018*
LDH, U/L	315 (129)	310 (130)	341 (100)	0.035*
ALT, U/L	31 (40)	30 (40.75)	32 (33)	0.948
AST, U/L	28 (29)	28 (30.75)	30 (19.5)	0.688
BUN, mmol/L	4.57 (2.49)	4.4 (2.50)	5.5 (2.65)	<0.001*
SCr, μmol/L	64.99 (23.75)	64.61 (24.00)	65.4 (29.49)	0.221
K, mmol/L	4 (0.64)	3.99 (0.66)	4.07 (0.51)	0.44
Na, mmol/L	137 (5.2)	137 (5.08)	137.3 (5.45)	0.617
Ca, mmol/L	2.19 (0.35)	2.21 (0.32)	1.91 (0.33)	<0.001*
FIB, g/L	4.16 (2.57)	3.98 (2.53)	4.67 (2.29)	0.001*
D_Dimer, mg/L	2.91 (2.10)	2.64 (1.94)	4.18 (1.25)	<0.001*

Count data presented as *n* (%) prevalence and compared using *χ*^2^-test.

Continuous variables are presented as mean ± standard deviation (Mean ± SD) for normally distributed data (Hb, TP, ALB) and compared using the independent samples *t*-test, or as median (interquartile range) [Median (IQR)] for non-normally distributed data and compared using the Mann–Whitney *U* test.

* *P-*value < 0.05 were considered statistically significant.

Reference ranges for laboratory parameters: CRP 0–10 mg/L; WBC 3.5–9.5 × 10^9^/L; N 1.8–6.3 × 10^9^/L; RBC 4.0–5.5 × 10^12^/L; Hb 120–160 g/L; PLT 125–350 × 10^9^/L; AMY 0–100 U/L; LiP 0–60 U/L; GLU 3.9–6.0 mmol/L; CHO 2.26–6.0 mmol/L; TG 0.7–1.7 mmol/L; TP 65–85 g/L; ALB 35–55 g/L; GLO 20–40 g/L; TBIL 0–26 μmol/L; DBIL 0–6.8 μmol/L; IBIL 1.4–13.5 μmol/L; ALP 45–125 U/L; LDH 90–245 U/L; ALT 0–42 U/L; AST 0–37 U/L; BUN 1.7–8.3 mmol/L; SCr 40–110 μmol/L; K 3.5–5.1 mmol/L; Na 137–147 mmol/L; Ca 2.13–2.9 mmol/L; FIB 2–4 g/L; D-Dimer 0–0.55 mg/L. All tests were performed using routine clinical laboratory methods at our institution.

### 3.2 Feature selection

**Figure 2:**
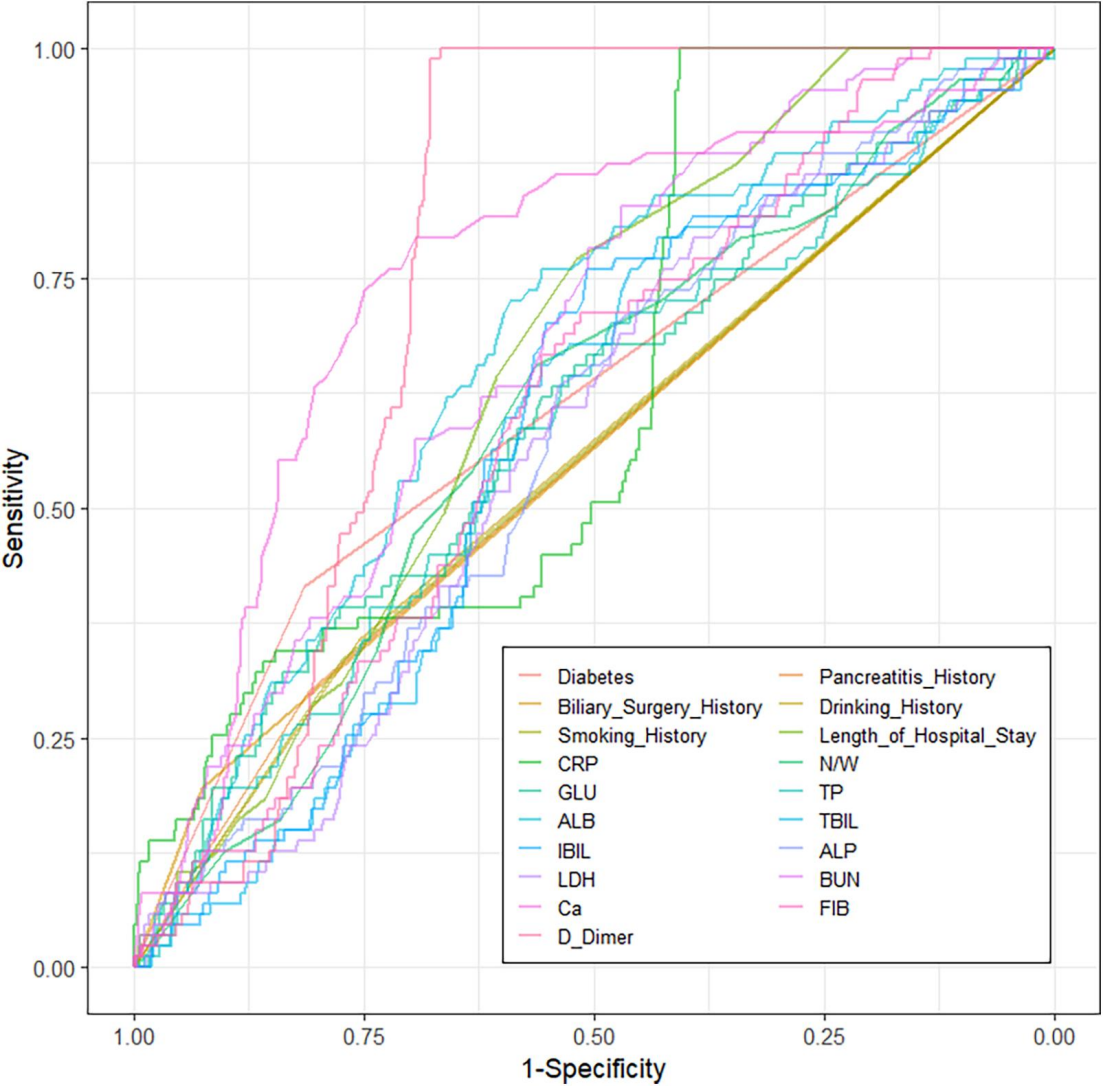
Univariate ROC curves.

**Figure 3:**
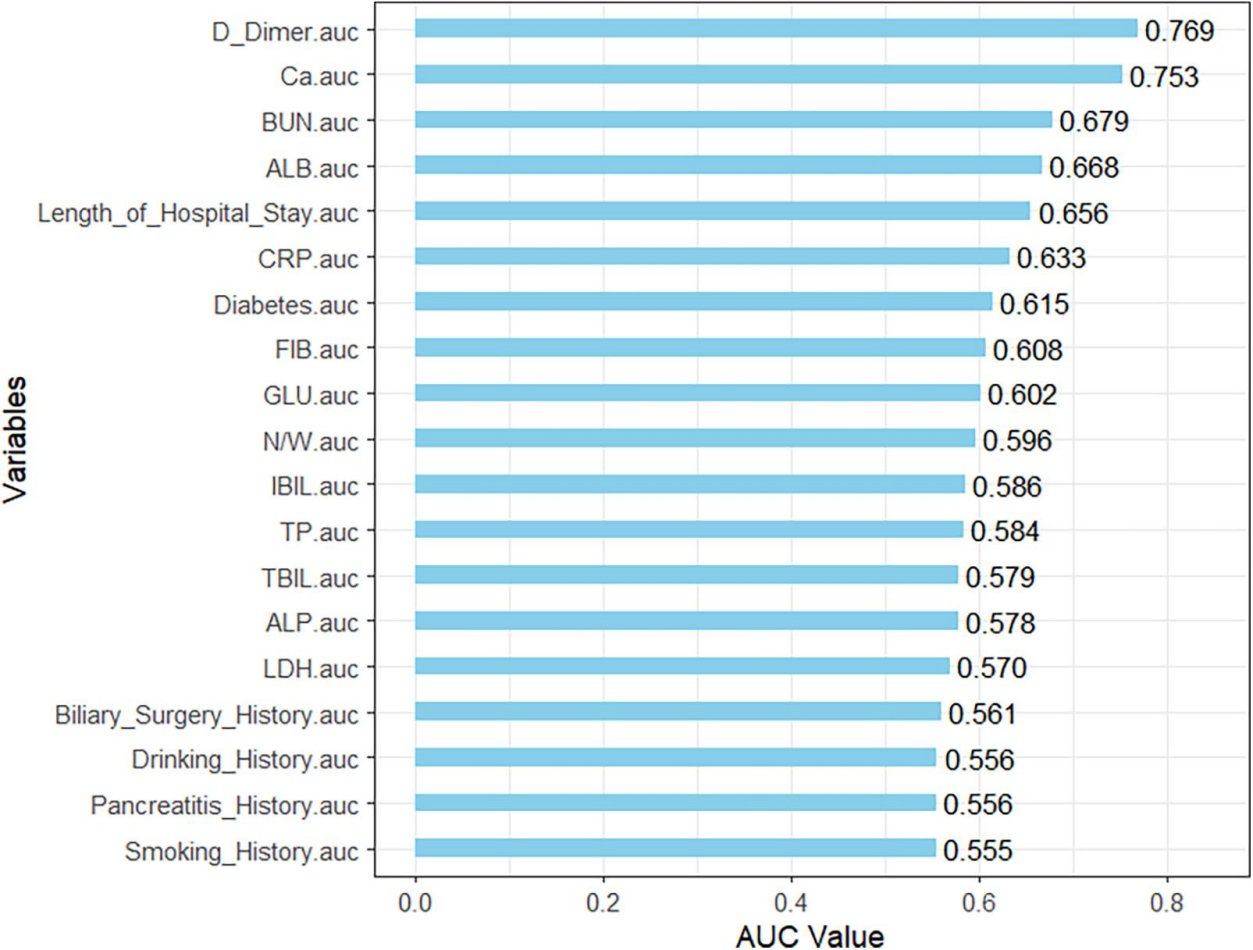
Bar plot of univariate AUC values.

**Figure 4:**
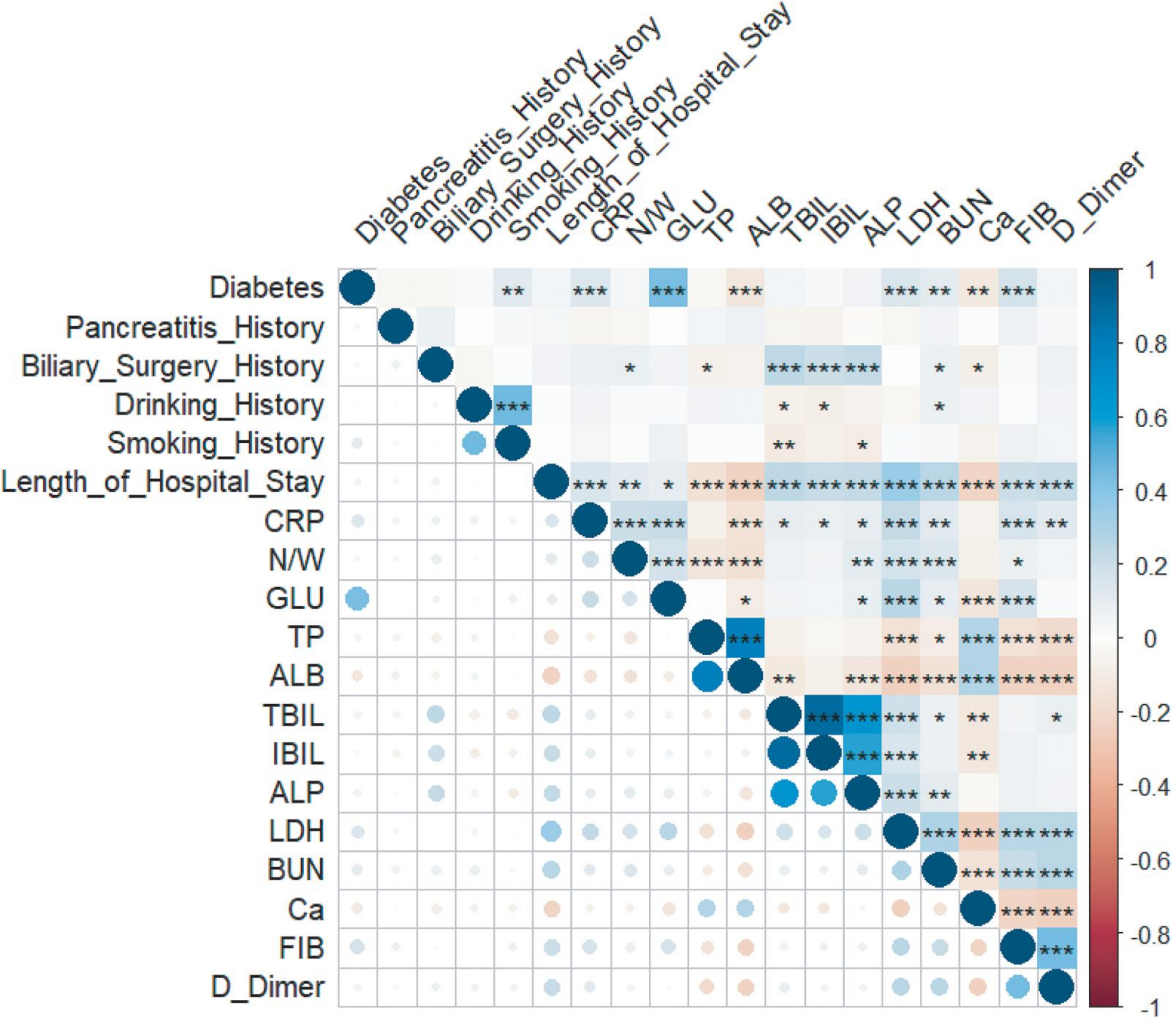
Variable correlation heatmap.

**Figure 5:**
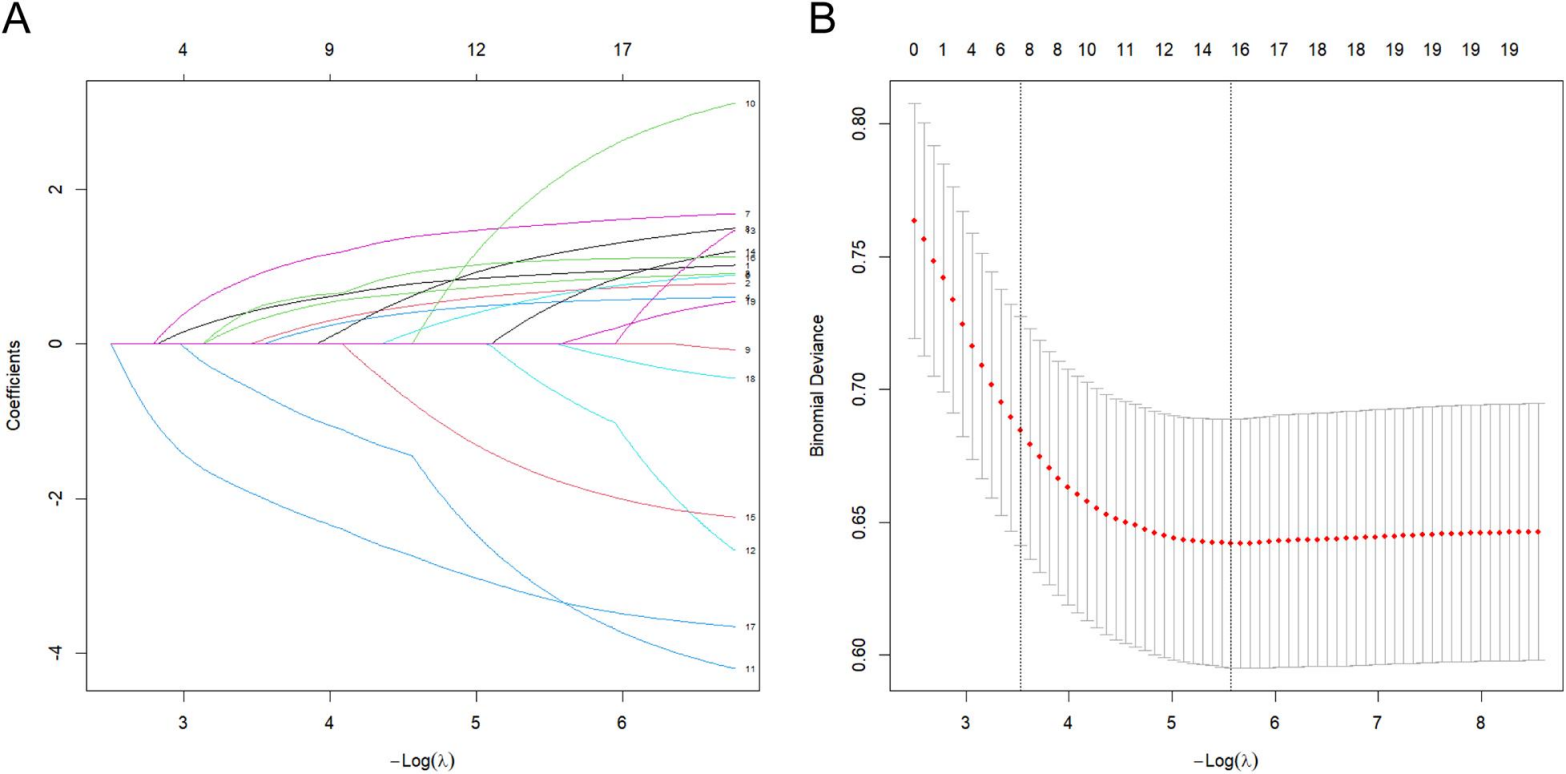
Feature selection using LASSO regression. **(A)** LASSO coefficient path plot. **(B)** LASSO cross-validation curve.

Using *P* < 0.05 as the threshold, 19 candidate variables were initially screened from the 45 initial variables. Univariate analysis showed D-Dimer had the most significant predictive power (AUC = 0.769) (Univariate ROC curves in Figure 2, AUC ranking bar plot in Figure 3). The correlation heatmap indicated strong correlations among these candidate variables (Figure 4). To further reduce dimensionality and address multicollinearity, LASSO regression was used for feature selection. The optimal penalty coefficient *λ* (lambda.1se) was determined via 20-fold cross-validation, and 7 variables with non-zero coefficients were identified for subsequent modeling: Diabetes History, Pancreatitis History, Biliary Surgery History, CRP, ALB, BUN, and Ca. Other variables such as Smoking History, Pleural Effusion, Days Since Onset, Length of Hospital Stay, BMI, and other laboratory indicators were compressed to zero and excluded from the final model (Figure 5).

### 3.3 Model performance

**Figure 6:**
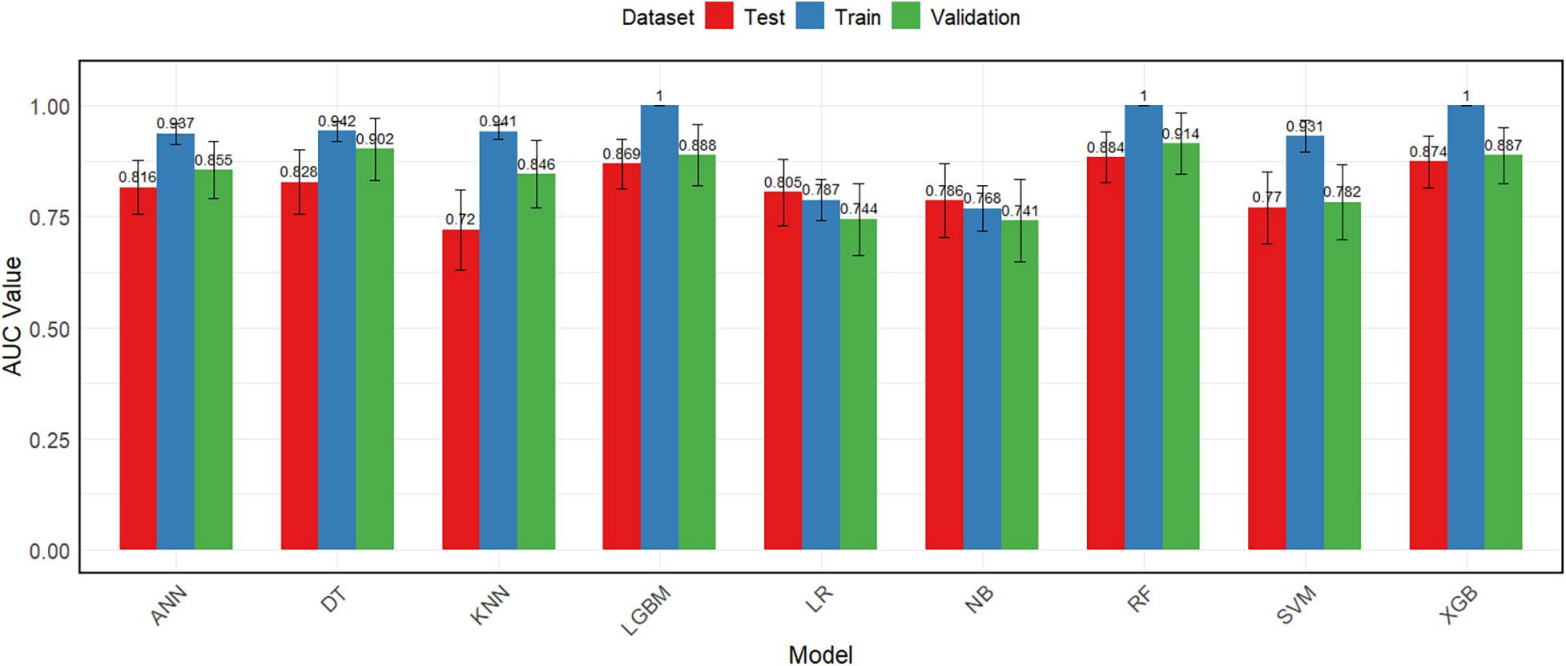
Bar chart comparing AUC of different models across the three datasets.

**Figure 7:**
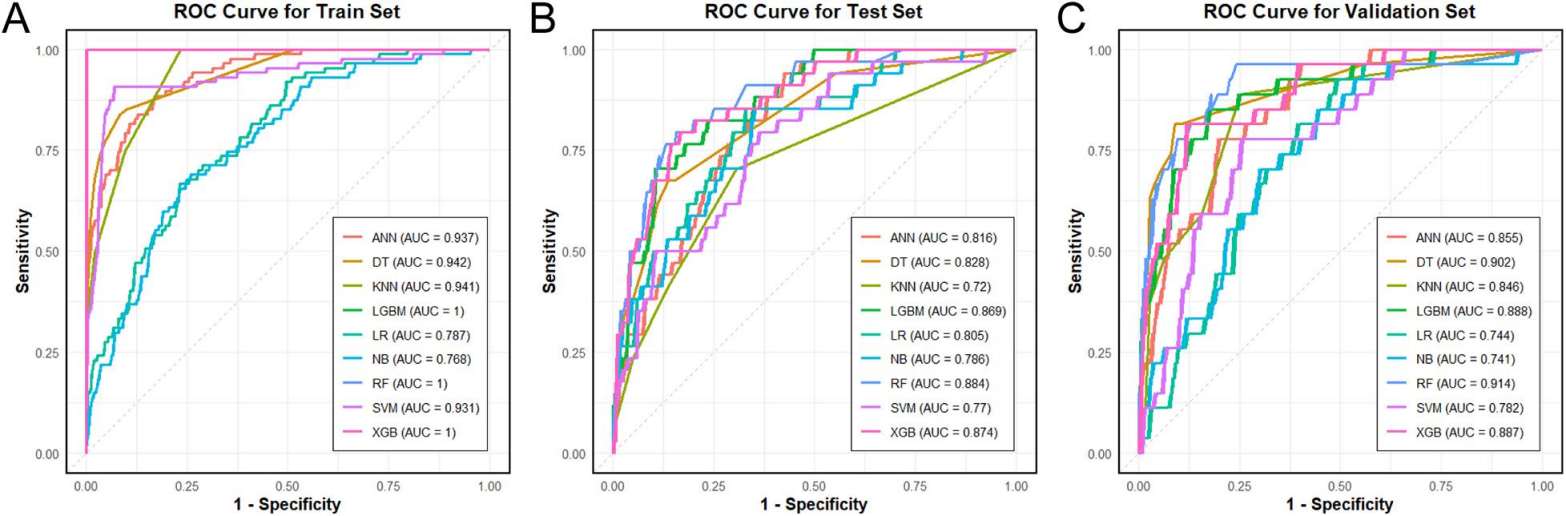
Comparison of ROC curves for different machine learning models across the three datasets. **(A)** Training set, **(B)** Test set, **(C)** Validation set.

The seven variables selected by LASSO regression (diabetes history, pancreatitis history, biliary surgery history, C-reactive protein, albumin, blood urea nitrogen, and serum calcium) were used as input features for all nine machine learning models. Table 3 presents the comprehensive performance metrics—including area under the receiver operating characteristic curve (AUC), accuracy, sensitivity, specificity, positive predictive value (PPV), and negative predictive value (NPV)—for each model on the training, internal test, and temporal validation sets, with corresponding 95% confidence intervals; Figures 6, 7 display the bar charts and ROC curves.

**Table 3:** Comprehensive performance metrics of All machine learning models across training, test, and temporal validation sets.

Model	Dataset	AUC	Accuracy	Sensitivity	Specificity	PPV	NPV
LR	Train	0.787 (0.741–0.834)	0.883 (0.857–0.905)	0.149 (0.089–0.239)	0.990 (0.978–0.995)	0.684 (0.460–0.846)	0.889 (0.863–0.911)
LR	Test	0.805 (0.729–0.880)	0.891 (0.850–0.922)	0.147 (0.064–0.301)	0.988 (0.967–0.996)	0.625 (0.306–0.863)	0.899 (0.858–0.928)
LR	Validation	0.744 (0.663–0.825)	0.868 (0.815–0.908)	0.037 (0.007–0.183)	0.994 (0.969–0.999)	0.500 (0.095–0.905)	0.872 (0.819–0.911)
DT	Train	0.942 (0.919–0.966)	0.942 (0.921–0.957)	0.678 (0.574–0.767)	0.980 (0.965–0.988)	0.831 (0.727–0.901)	0.954 (0.935–0.968)
DT	Test	0.828 (0.755–0.901)	0.871 (0.828–0.904)	0.471 (0.315–0.633)	0.923 (0.884–0.950)	0.444 (0.295–0.604)	0.930 (0.892–0.955)
DT	Validation	0.902 (0.832–0.971)	0.922 (0.877–0.951)	0.667 (0.478–0.814)	0.961 (0.921–0.981)	0.720 (0.524–0.857)	0.950 (0.908–0.973)
RF	Train	1.000 (1.000–1.000)	0.999 (0.992–1.000)	0.989 (0.938–0.998)	1.000 (0.994–1.000)	1.000 (0.957–1.000)	0.998 (0.991–1.000)
RF	Test	0.884 (0.827–0.941)	0.901 (0.862–0.930)	0.353 (0.215–0.521)	0.973 (0.945–0.987)	0.632 (0.410–0.809)	0.920 (0.882–0.947)
RF	Validation	0.914 (0.845–0.983)	0.912 (0.865–0.944)	0.481 (0.307–0.660)	0.978 (0.944–0.991)	0.765 (0.527–0.904)	0.926 (0.879–0.955)
ANN	Train	0.937 (0.913–0.961)	0.928 (0.907–0.945)	0.517 (0.414–0.619)	0.988 (0.976–0.994)	0.865 (0.747–0.933)	0.934 (0.912–0.951)
ANN	Test	0.816 (0.755–0.877)	0.874 (0.831–0.907)	0.294 (0.168–0.462)	0.950 (0.916–0.971)	0.435 (0.256–0.632)	0.911 (0.872–0.940)
ANN	Validation	0.855 (0.790–0.920)	0.868 (0.815–0.908)	0.407 (0.245–0.593)	0.938 (0.893–0.965)	0.500 (0.307–0.693)	0.913 (0.863–0.945)
SVM	Train	0.931 (0.896–0.966)	0.904 (0.879–0.924)	0.264 (0.183–0.366)	0.997 (0.988–0.999)	0.920 (0.750–0.978)	0.903 (0.878–0.923)
SVM	Test	0.770 (0.689–0.851)	0.888 (0.847–0.919)	0.147 (0.064–0.301)	0.985 (0.961–0.994)	0.556 (0.267–0.811)	0.898 (0.858–0.928)
SVM	Validation	0.782 (0.698–0.867)	0.868 (0.815–0.908)	0.074 (0.021–0.234)	0.989 (0.960–0.997)	0.500 (0.150–0.850)	0.876 (0.823–0.914)
KNN	Train	0.941 (0.923–0.958)	0.917 (0.894–0.935)	0.494 (0.392–0.597)	0.978 (0.963–0.987)	0.768 (0.642–0.859)	0.930 (0.907–0.947)
KNN	Test	0.720 (0.629–0.811)	0.867 (0.824–0.901)	0.235 (0.124–0.400)	0.950 (0.916–0.971)	0.381 (0.208–0.591)	0.905 (0.864–0.934)
KNN	Validation	0.846 (0.769–0.922)	0.878 (0.826–0.916)	0.481 (0.307–0.660)	0.938 (0.893–0.965)	0.542 (0.351–0.721)	0.923 (0.874–0.953)
NB	Train	0.768 (0.718–0.819)	0.834 (0.804–0.860)	0.333 (0.243–0.438)	0.906 (0.880–0.927)	0.341 (0.249–0.447)	0.903 (0.877–0.924)
NB	Test	0.786 (0.703–0.869)	0.864 (0.820–0.898)	0.412 (0.264–0.578)	0.923 (0.884–0.950)	0.412 (0.264–0.578)	0.923 (0.884–0.950)
NB	Validation	0.741 (0.648–0.833)	0.854 (0.799–0.896)	0.222 (0.106–0.408)	0.949 (0.907–0.973)	0.400 (0.198–0.643)	0.889 (0.837–0.927)
XGB	Train	1.000 (1.000–1.000)	1.000 (0.994–1.000)	1.000 (0.958–1.000)	1.000 (0.994–1.000)	1.000 (0.958–1.000)	1.000 (0.994–1.000)
XGB	Test	0.874 (0.815–0.932)	0.898 (0.858–0.928)	0.471 (0.315–0.633)	0.954 (0.921–0.973)	0.571 (0.391–0.735)	0.932 (0.896–0.957)
XGB	Validation	0.887 (0.824–0.951)	0.883 (0.832–0.920)	0.519 (0.340–0.693)	0.938 (0.893–0.965)	0.560 (0.371–0.733)	0.928 (0.880–0.957)
LGBM	Train	1.000 (1.000–1.000)	1.000 (0.994–1.000)	1.000 (0.958–1.000)	1.000 (0.994–1.000)	1.000 (0.958–1.000)	1.000 (0.994–1.000)
LGBM	Test	0.869 (0.814–0.925)	0.895 (0.854–0.925)	0.471 (0.315–0.633)	0.950 (0.916–0.971)	0.552 (0.375–0.716)	0.932 (0.895–0.957)
LGBM	Validation	0.888 (0.819–0.957)	0.883 (0.832–0.920)	0.556 (0.373–0.724)	0.933 (0.886–0.961)	0.556 (0.373–0.724)	0.933 (0.886–0.961)

On the training set, the Random Forest (RF) model achieved an AUC of 1.000 (95% CI: 1.000–1.000), accuracy of 0.999 (0.992–1.000), sensitivity of 0.989 (0.938–0.998), specificity of 1.000 (0.994–1.000), PPV of 1.000 (0.957–1.000), and NPV of 0.998 (0.991–1.000). On the internal test set, RF yielded an AUC of 0.884 (0.827–0.941), accuracy of 0.901 (0.862–0.930), sensitivity of 0.353 (0.215–0.521), specificity of 0.973 (0.945–0.987), PPV of 0.632 (0.410–0.809), and NPV of 0.920 (0.882–0.947). On the temporal validation set, RF achieved an AUC of 0.914 (0.845–0.983), accuracy of 0.912 (0.865–0.944), sensitivity of 0.481 (0.307–0.660), specificity of 0.978 (0.944–0.991), PPV of 0.765 (0.527–0.904), and NPV of 0.926 (0.879–0.955).

Among the other models, XGBoost and LightGBM also attained perfect training AUCs (1.000) and maintained validation AUCs above 0.88, with specific metrics detailed in Table 2. Decision Tree (DT) showed training, test, and validation AUCs of 0.942, 0.828, and 0.902, respectively. K-Nearest Neighbors (KNN) exhibited a training AUC of 0.941, test AUC of 0.720, and validation AUC of 0.846. Artificial Neural Network (ANN) yielded AUCs of 0.937 (training), 0.816 (test), and 0.855 (validation). Support Vector Machine (SVM) produced AUCs of 0.931 (training), 0.770 (test), and 0.782 (validation). Logistic Regression (LR) and Naïve Bayes (NB) demonstrated more stable but lower AUCs across datasets, with validation AUCs of 0.744 and 0.741, respectively.

**Figure 8:**
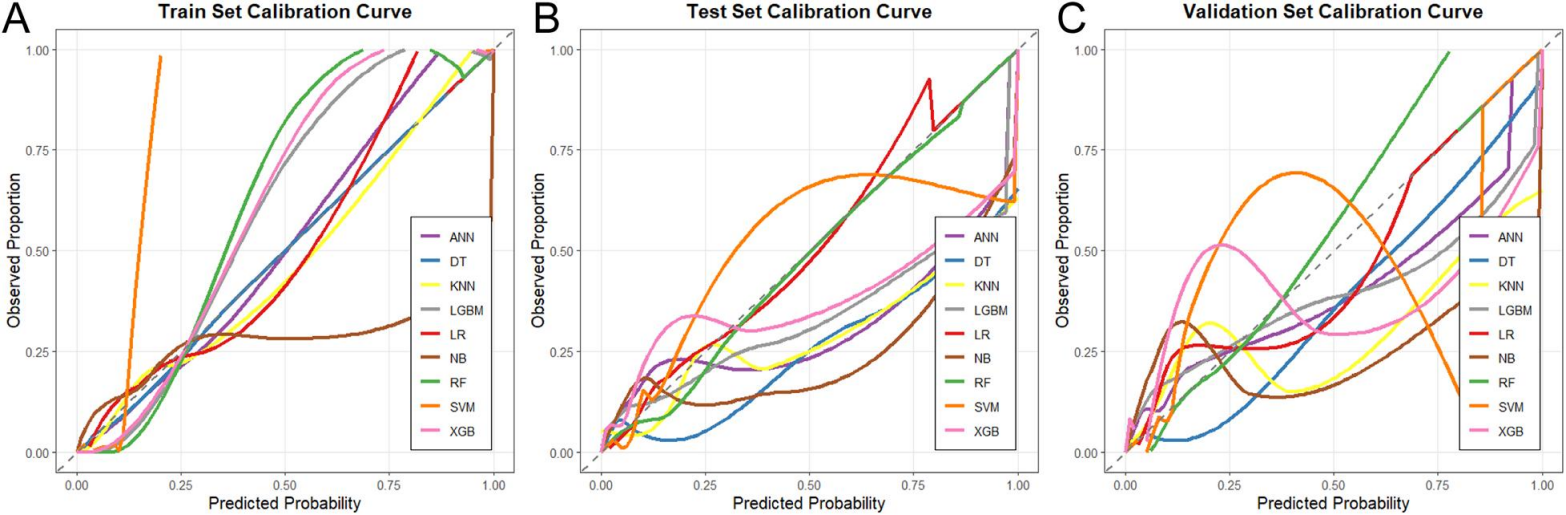
Calibration curves for different machine learning models across the three datasets. **(A)** Training set, **(B)** Test set, **(C)** Validation set.

Calibration curves were plotted to assess the agreement between predicted probabilities and observed outcomes for all nine models across the three datasets (Figure 8). The Random Forest model demonstrated good calibration in both the test and validation sets, with predictions closely aligning with the ideal diagonal line. Overall, the Random Forest model consistently achieved the highest AUC across all three datasets with well-balanced performance across other metrics, supporting its selection as the optimal model for further analysis.

### 3.4 Model interpretability

**Figure 9:**
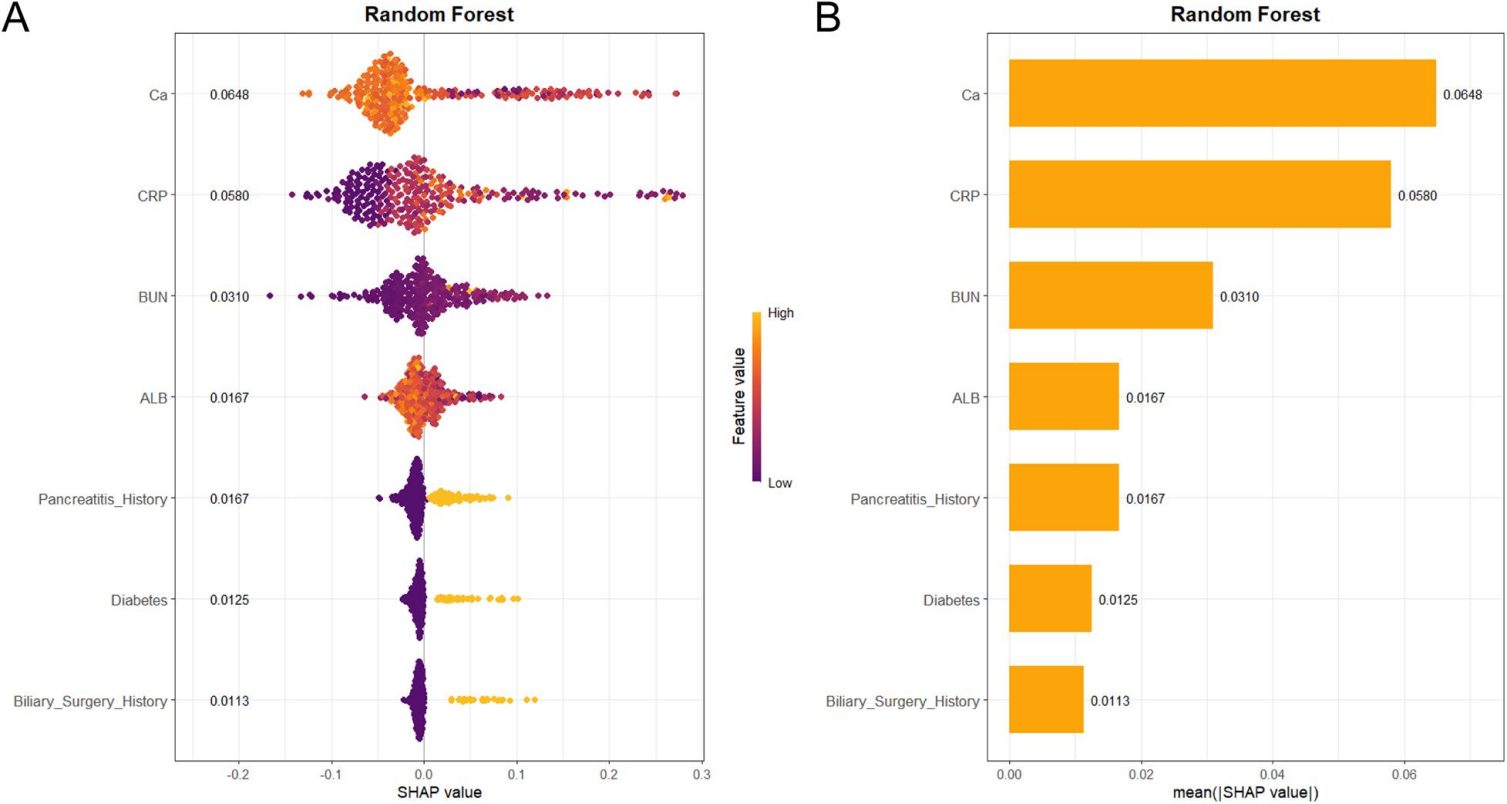
SHAP interpretability analysis based on the random forest model. **(A)** SHAP bee swarm plot. **(B)** Global feature importance ranking.

**Figure 10:**
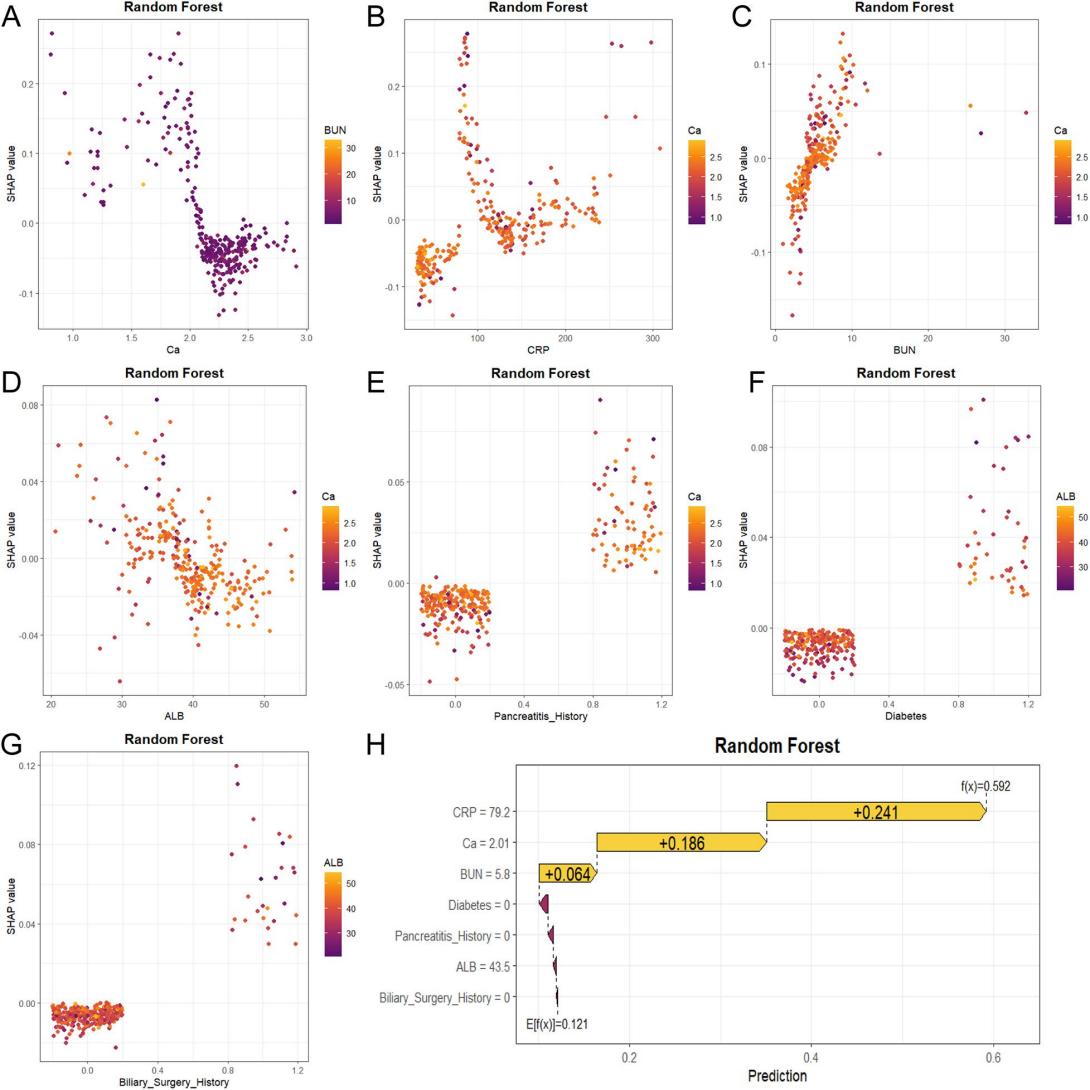
SHAP dependence plots for the top 7 features determining the risk of pancreatic pseudocyst in the training cohort. The features, in order of importance, are: Calcium **(A)**, C-reactive Protein **(B)**, Blood Urea Nitrogen **(C)**, Albumin **(D)**, Pancreatitis History **(E)**, Diabetes **(F)**, and Biliary Surgery History **(G)** Panels A-G illustrate how the value of each feature influences the model's output (SHAP value). Subplot **(H)** presents an individual prediction explanation for a specific instance (Patient #5 from the test set) using the Random Forest model. SHAP, Shapley Additive exPlanations. Based on the Youden index optimal threshold of 0.193, this patient was classified as high risk for developing pancreatic pseudocyst.

Based on the Random Forest model, the SHAP (SHapley Additive exPlanations) framework was applied to interpret both global feature importance and individual prediction behavior. The SHAP bee swarm plot (Figure 9A) illustrates how feature values influence model output: elevated values (shown in yellow) correspond to positive SHAP values, increasing the predicted risk of pancreatic pseudocyst (PPC), while lower values (purple) are associated with negative SHAP values, decreasing the risk—for instance, high CRP and low serum calcium levels substantially elevate PPC risk. Consistent with this, the global feature importance ranking (Figure 9B) identified serum calcium (Ca), CRP, blood urea nitrogen (BUN), albumin (ALB), and history of pancreatitis as the top five predictors. Furthermore, SHAP dependence plots (Figure 10A–G) detail the specific functional relationships between these key features and model output, excluding the influence of other variables. To illustrate the model's decision logic at an individual level, Patient #5 from the test set was randomly selected for explanation (Figure 10H). Starting from the baseline prediction risk (E[f(x)] = 0.121), a high CRP level (79.2 mg/L) contributed the largest risk increase (+0.241), and a low serum calcium level (2.01 mmol/L) further raised the risk significantly (+0.186), ultimately pushing the individual prediction (f(x) = 0.592) above the decision threshold. Based on the Youden index optimal threshold of 0.193, this patient was classified as high risk for developing pancreatic pseudocyst.

## 4 Discussion

Pancreatic pseudocyst (PPC) is a late local complication of acute pancreatitis (AP) that, if not detected and managed in a timely manner, can lead to life-threatening secondary events such as infection, hemorrhage, rupture, or obstruction, while also prolonging hospital stay and increasing medical costs (3, 21). However, current predictive research in this field remains largely limited to traditional logistic regression and nomogram models, and generally lacks rigorous temporal validation, hindering clinical translation (5, 6, 8). To address these limitations, this study developed prediction models using nine diverse machine learning algorithms based on clinical data from 1,184 AP patients. The algorithms included logistic regression (as a linear baseline), tree-based models (decision tree, random forest), ensemble methods (XGBoost, LightGBM), instance-based learning (K-nearest neighbors), kernel methods (support vector machine), probabilistic models (naïve Bayes), and neural networks (artificial neural network). This comprehensive selection was designed to evaluate models with different inductive biases: logistic regression for linearity and interpretability; tree-based and ensemble methods for capturing non-linear interactions and robustness; support vector machine for high-dimensional space separation; and neural networks for complex pattern recognition (9). Each model presents distinct trade-offs in terms of interpretability, ability to handle non-linear relationships, and risk of overfitting. Our comparative approach aimed to identify the optimal balance for this clinical classification task (22, 23).

A comprehensive comparative analysis of the performance metrics across all machine learning models revealed significant differences among the evaluated algorithms. Several methods, including Decision Tree (DT), K-Nearest Neighbors (KNN), Artificial Neural Network (ANN), and Support Vector Machine (SVM), exhibited signs of overfitting, with high training AUC values (above 0.93) but substantially degraded performance on both the independent test set and the temporal validation set. Among these, KNN showed the most pronounced decline, achieving a test AUC of only 0.720. Meanwhile, Logistic Regression (LR) and Naïve Bayes (NB) demonstrated consistent stability across datasets, with minimal performance gaps between the training, test, and validation sets; however, their overall discriminative ability remained limited, yielding validation AUC values around 0.74. In contrast, ensemble methods such as Random Forest (RF), XGBoost, and LightGBM attained near-perfect performance on the training set (AUC = 1.0), which may initially raise concerns about overfitting. This outcome can be attributed to the strong learning capacity of tree-based ensemble methods on the selected feature set and their ability to partition the training data with high precision (14). Crucially, all three ensemble models maintained robust and superior discriminative performance on both the test and temporal validation sets (AUC > 0.86), confirming their effective generalization capabilities. Meanwhile, in the comparison of other performance indicators, the random forest model performed well in terms of specificity, positive predictive value (PPV), and negative predictive value (NPV), but its sensitivity was average. Overall, Random Forest achieved the optimal balance between discriminative power and generalization, delivering the best comprehensive performance. This result underscores the advantage of ensemble learning approaches in capturing complex, non-linear relationships among clinical predictors while maintaining robustness on unseen data (10).

The 7 key predictive variables selected by LASSO regression—Ca, CRP, BUN, ALB, Pancreatitis History, Diabetes History, and Biliary Surgery History—comprehensively cover multiple pathological dimensions including inflammation, metabolism, nutritional status, and pancreatic structure. Low serum calcium reflects the severity of fat necrosis and saponification, a key marker of severe AP and local complications (18, 24). CRP, as a key systemic inflammation indicator, its elevation is closely related to pancreatic and peripancreatic necrosis (8). In AP, an elevated BUN signals renal impairment, which can be caused by both the systemic inflammatory response and direct vascular compression from pseudocysts, the latter posing a threat of thrombosis and significant renal injury (25, 26). A history of acute pancreatitis often indicates potential pancreatic duct abnormalities, and longer delays in seeking treatment in these patients further increase complication risk (4, 27, 28). Hypoalbuminemia reflects both malnutrition and inflammatory consumption, affecting tissue repair and fluid absorption (29, 30). In AP patients with diabetes, insufficient insulin secretion and outflow obstruction lead to further elevated blood glucose, exacerbating pancreatic damage and delaying inflammation resolution and tissue repair (31, 32). Biliary surgery alters biliopancreatic duct anatomy, potentially causing strictures and increasing PPC risk (5). Importantly, SHAP analysis revealed that these features do not contribute equally; serum calcium (Ca) and CRP were the top two drivers, followed by BUN, ALB, and Pancreatitis History. This differential contribution provides a hierarchy for clinical attention and highlights the importance of acute inflammatory and metabolic disturbances in PPC development.

These factors collectively inform a multi-mechanistic framework for understanding PPC development and provide a basis for early identification of high-risk patients. Based on the key predictive variables identified by the model, prevention of PPC following acute pancreatitis may benefit from a stratified management approach integrating early warning, multi-target intervention, and long-term follow-up. Rapid risk assessment should be initiated upon admission. The model's predictions could help guide the intensity and focus of subsequent management and follow-up. For example, clinicians might be prompted to intensify monitoring and consider early preventive interventions for patients identified as high-risk, such as Patient #5 from the test set. Patients with high-risk indicators (e.g., Ca < 2.0 mmol/L, CRP > 150 mg/L, ALB < 30 g/L, or a history of pancreatitis) may warrant intensified monitoring. Early measures include cautious fluid resuscitation with Lactated Ringer's solution to correct electrolyte imbalances and maintain microcirculation while avoiding over-infusion (33). Short-term use of anti-inflammatory drugs may be considered for those with markedly elevated inflammatory markers. Regarding nutritional support, an oral low-fat diet should be initiated as early as tolerated; when oral intake is not feasible, enteral nutrition should be started within 24–48 h, with albumin supplementation for patients with hypoalbuminemia (34, 35). Metabolic management includes maintaining normal glucose homeostasis through appropriate blood glucose control (36). For patients with excessive pancreatic secretion or suspected pancreatic duct abnormalities, extending the duration of somatostatin analogue therapy may be considered to further suppress exocrine activity and potentially mitigate pseudocyst formation (37). Long-term management includes alcohol cessation counseling, lipid control, and regular imaging follow-up (contrast-enhanced CT or MRI at 4–8 weeks after discharge, repeated every 3–6 months thereafter) (38). This multidimensional, staged approach may contribute to reducing PPC risk and improving patient outcomes. It should be noted, however, that risk classification is not absolute, and PPC may still occur in patients identified as low risk.

This study has several methodological strengths. First, LASSO regression was used for feature selection, offering advantages over traditional stepwise regression. By introducing an L1 regularization penalty, it automates variable selection while preserving model interpretability, effectively addressing multicollinearity and enhancing generalizability (39). Second, nine mainstream machine learning algorithms were systematically compared; Random Forest demonstrated the best performance on both the test and temporal validation sets (AUCs 0.884 and 0.914, respectively), indicating robust generalization. As an ensemble method, Random Forest improves predictive accuracy and stability through a “voting” mechanism across multiple decision trees, underscoring the value of ensemble approaches in medical prediction tasks (13). Third, the use of an independent temporal validation set (2023 data) simulates model performance on a new patient cohort, strengthening the credibility of its clinical applicability. Compared with studies relying solely on internal cross-validation, this design offers a more prospective and practical assessment. Fourth, the incorporation of SHAP for interpretability analysis not only quantifies each feature's contribution but also visually illustrates the direction and nonlinear nature of feature effects through bee swarm plots, dependence plots, and individual sample explanations. This enhances transparency and may facilitate clinical acceptance (40, 41).

Several limitations must be acknowledged. First, the retrospective, single-center design may introduce selection bias and limit the generalizability of the findings across different institutions and populations. Although the 2023 cohort served as a temporal validation set to assess model stability over time, this approach cannot guarantee generalizability to other centers, patient populations, or clinical settings. True external validation using independent multi-center data remains a critical direction for future research. Additionally, a total of 889 cases were excluded, primarily due to incomplete follow-up or missing key data, which could affect the representativeness of the cohort. Reliance on historical medical records may also lead to issues such as incomplete documentation, inconsistent data recording, or measurement errors, potentially compromising the reliability of the model. Second, factors such as acute pancreatitis severity classification, organ failure, and the CT severity index (CTSI) are also closely associated with PPC development, and the omission of these factors may introduce unmeasured confounding. Furthermore, in acute pancreatitis, biomarkers such as calcium, C-reactive protein, blood urea nitrogen, and albumin can fluctuate rapidly during the early disease course. Relying solely on initial values obtained at admission may not fully capture their dynamic changes and could limit the clinical applicability of the model. Future research should aim to validate and refine this model through prospective, multicenter, large-scale cohort studies involving broader populations. Integrating multi-omics data and dynamic monitoring indicators could further enhance the robustness and precision of the risk classification system, ultimately promoting personalized and precision management of acute pancreatitis complications.

## 5 Conclusions

This study successfully developed and validated an interpretable machine learning model for PPC risk classification using seven routinely available clinical indicators. The random forest model demonstrated strong discriminative performance and generalizability, while SHAP analysis provided transparent explanations of individual predictions. By enabling early identification of high-risk patients, this model may support clinical decision-making and improve management of AP complications.

## Data Availability

The raw data supporting the conclusions of this article will be made available by the authors, without undue reservation.
